# Systemic Racism in EEG Research: Considerations and Potential Solutions

**DOI:** 10.1007/s42761-021-00050-0

**Published:** 2021-05-26

**Authors:** Tricia Choy, Elizabeth Baker, Katherine Stavropoulos

**Affiliations:** Graduate School of Education, University of California, Riverside, 1207 Sproul Hall, Riverside, CA 95251 USA

**Keywords:** Systemic racism, EEG, Neuroscience, Minority samples

## Abstract

The current theoretical paper discusses the unintended systemic racism and racial biases that impact neuroscience, specifically in research utilizing electroencephalography (EEG). As a popular technique in affective science research, EEG requires adherence between the electrode and scalp to measure brain activity. To obtain high-quality data, various factors such as hair length, hair type, body movement, and/or extraneous noise from the environment are taken into consideration. As EEG researchers attempt to gather good-quality data, the recruitment and retention of Black American participants is challenging due to hairstyles commonly worn by Black American participants (e.g., cornrows, braids) and hair type. Taken together, the systemic lack of data from Black American participants renders research findings less generalizable and causes disparities in theoretical knowledge applicable to this population. To address this disparity, innovative solutions invented by bioengineers are discussed.

## Neuroscience Overview

Neuroscience research has improved our understanding of how brain function and behavior are interconnected, and has also provided important information about neural mechanisms of a variety of cognitive and affective processes. Non-invasive brain imaging techniques are the most common methods utilized to study brain activity and brain function. These techniques include electroencephalography (EEG), magnetoencephalography (MEG), positron-emission tomography (PET), functional magnetic resonance imaging (fMRI), and functional near-infrared spectroscopy (fNIRS). These techniques are widely used to capture brain development and activity across the life span from infants to adults. In the context of affective science, EEG has been utilized to study affective processes (e.g., neural correlates of face processing, the effect of emotional stimuli on memory encoding). For example, to gain insight into the relationship of arousal, memory, and emotional stimuli, Zarubin et al. (2020) utilized EEG methodology to investigate how passively viewing negative and neutral images related to the encoding of emotional memories. Nevertheless, neuroscientific research is not without shortcomings and it is important to acknowledge aspects of systemic racism and racial bias that exist within these methods. Although EEG is a popular technique in affective neuroscience research, this position paper focuses on the inadvertent exclusion of certain minority populations due to hairstyles and hair type, thus leading to biases and inability to generalize findings.

## Issues of Systemic Bias in Neuroscience: Recruitment and Retention

To provide conceptual evidence of the existing disparities in the inclusion of minority races and ethnicities in EEG literature, a search was conducted between the months of September 2019 to October 2019 using PubMed, EBSCOhost, and JSTOR. Key terms searched included EEG, neurotypical adult or adults, and variations of minority populations (e.g., minority sample, ethnicity, or racial minority). Search results included a total of 81 peer-reviewed articles. Of these 81 articles, only five articles explicitly reported having a sample with Black American participants, but it was unclear if these participants’ data were included in final analyses. Similarly, only four papers included literature reviews on the recruitment and retention of historically minoritized participants. Thus, this literature search was not conducted to be systematic or comprehensive, but to provide a proof of concept of the existing disparities in the inclusion of minoritized races and ethnicities in neuroscience research. Fortunately, the research community recognizes disproportionate issues related to recruitment of diverse and minority samples within neuroscience research and has explored potential reasons for this phenomenon.

Research reviews have been conducted to study the barriers and motivators as well as recruitment and retention methods of minority populations (e.g., African Americans, Asian Americans, Pacific Islanders, and Latinos) within neuroscience research (George et al., 2014; Habibi et al., 2015). Previous literature has also discussed the feasibility of using images rather than written descriptions during recruitment to increase potential participants’ understanding of neuroscience research methods (e.g., Losh et al., 2020). Thus, the literature contains acknowledgement of the need for increased inclusion of ethnic minority communities in neuroscience research to improve the representation of minority subgroups and generalizability of research findings. Despite this recognition, due to the nature of collecting and measuring brain activity, there has been an unforeseen intersectionality between EEG research and systemic racism.

## Issues of Systemic Bias in Neuroscience: EEG and Exclusionary Criteria

Depending on the focus of the study, certain neuroscience techniques may be utilized to achieve the best measure of brain activity while also adhering to procedural guidelines. Human brain imaging techniques are divided into two categories—metabolic-based (fMRI, PET, fNIRS) and electrophysiological-based (EEG and MEG). Metabolic-based brain imaging is known to have good spatial resolution and poor temporal resolution; in contrast, electrophysiological-based brain imaging is known to have poor spatial resolution and exceptional temporal resolution (Burle et al., 2015). Spatial resolution refers to *where* brain activity is occurring, while temporal resolution corresponds to *when* brain activity is occurring. EEG is a relatively inexpensive technology where participants wear a cap with attached electrodes to measure brain activity. As previously mentioned, EEG has exceptional temporal resolution and involves the recording, analysis, and interpretation of voltages recorded on the scalp. During data collection, participants are prepped by measuring and fitting their head circumference to an EEG cap and saline (e.g., liquid and/or gel) solution is added to the electrodes. Using a saline solution is a common way to conduct electrical signals from the surface of the scalp to the electrode. Event-related potentials (ERPs) are measures of activity in response to presented stimuli and allow researchers to measure neural responses that are associated with specific sensory, cognitive, and motor events (Luck, 2005). Because it is important for electrodes to adhere to the scalp to record acceptable data for the analysis of ERPs, certain characteristics of hairstyles and hair type are taken into consideration for data collection. Thus, this position paper will focus on how EEG research has inadvertently excluded minority participants due to their hairstyles and hair type.

Certain hairstyles worn predominantly by Black American participants not only impact the quality of data collected, but there are also general guidelines that influence recruitment and retention of Black American individuals. In EEG studies, short and/or thin hair is ideal to achieve optimal measurement with proper adherence between the electrode, saline solution, and the scalp. Using objective measurements of hair curvature and hair curliness, participants from African, African American, and Caribbean regions of origin were found to have the highest classification for curly and tightly coiled hair (Loussouarn et al., 2007; Takahashi, 2019). Thus, hair types characterized as curly and tightly coiled hair can be developed into a variety of hairstyles, such as braids or cornrows, which may impede proper adherence to the scalp and the recording of EEG data. Many EEG labs ask participants if they have cornrows or braids, and if participants answer in the affirmative, then researchers ask if they are comfortable with removing those hairstyles prior to participation. If they disagree, they are often unable to participate because these hairstyles and hair type are considered exclusionary due to poor adherence between the electrode and scalp. More specifically, because cornrows, braids, and curly, tight-coiled hair are either closely braided against the scalp or left natural, it is not possible to obtain proper adherence (i.e., direct contact between the scalp, saline solution, and electrode to reduce extraneous noise and resistance) when collecting EEG data, which will be discussed further in the next section.

## Issues of Systemic Bias in Neuroscience: EEG and Data Quality

In a study of ethnic hair, Franbourg et al. (2003) found that in comparison to Asian and Caucasian hair, African hair characteristics included frequent twists with random reversals in directions and pronounced flattening, or a collapsed structure in the region of the twist. The natural characteristics of African hair impact the ability of EEG caps and saline solution to conduct brain activity, as there must be a direct connection between the scalp, saline solution, and EEG electrode(s). Additionally, while testing for hair absorption, African hair appeared to have the lowest radial swelling rate and lower swelling percentage in water which was measured through variation of diameter of the hair fiber compared to Asian and Caucasian hair (Franbourg et al., 2003). That is, African hair is less likely to absorb liquid compared to Asian and Caucasian hair. Due to this lack of hair absorption of African hair, it may be that the saline solution is unable to act as a proper conductor or too much of the saline solution is left near the electrode obstructing the ability to conduct neural responses from the scalp into the electrode attached to the EEG cap. Therefore, when Black American participants are willing to leave their hair in its natural state (e.g., remove cornrows or braids), there is a disproportionality with data retention which will be discussed in further detail later on in this section of the paper.

There are parameters commonly used during the data collection phase of EEG research studies, regarding acceptable signal-to-noise (S/N) ratios and impedance thresholds, that impact the retention of Black American participants. S/N ratio refers to the signal of the brain activity detected through the electrode in relation to the extraneous noise from the surrounding environment (e.g., electrical wires, lights, computer monitor, and electrical currents from cellphones) and skin of the participant. To reduce noise from the environment and boost the available brain activity “signal,” experimenters typically clean and/or abrade (e.g., adding an exfoliant or sandy cleanser) the skin, and may add saline solutions between the skin and electrode. Correspondingly, electrode impedance is the alternating current flow between electrodes in relation to the highly conductive skin tissue and overall brain activity (Kappenman & Luck, 2010). Strict impedance thresholds and a high S/N ratio may limit the inclusion of Black American participants on the basis of hair type. For example, meeting low impedance microvolt (μV) standards may be difficult due to curliness and tightly coiled hair, which are defining features of African hair. If impedance thresholds are not met, this then lowers the S/N ratio, which makes collected data less likely to meet the stringent requirements of acceptable and useable data during the data processing phase. As such, fewer Black American participants’ data may be included in statistical analyses, thus reducing generalizability of EEG findings while also disproportionately excluding Black American participants.

One method to improve the quality of brain activity recording is through the use of different electrode types. To understand the effects of different types of electrodes and their impact on electrode-skin impedance, Li et al. (2017) assessed wet, semi-dry, and dry electrodes on different areas of the body (e.g., hairy scalp, forehead, and forearm locations) and measured electrode-skin impedance in three different conditions—normal, pressure, and skin abrasion. Wet electrodes were made of silver/silver chloride (Ag/AgCl) materials with conductive gel; the semi-dry electrodes were five porous ceramic pillars and an Ag/AgCl electrode inside a 500-microliter (μL) saline reservoir; and the dry electrodes had eight conductive flexible plastic tips and a snap connector using passive design. Wet electrodes are the typical “gold standard” for clinics and laboratories for EEG recording due to good S/N ratio and high reliability. In more recent years, there have been developments in dry electrodes where electronic conductors without gel are placed on metallic tips, comb-like conductive polymer elastomers, and flexible-coated bristles (Grozea et al., 2011; Kitoko et al., 2011). Because no gel is required for dry electrode setup, there are many benefits to the utilization of dry electrodes, such as their cleanliness, quick setup, user friendliness, and self-application (Li et al., 2017). However, some disadvantages of dry electrodes include not conforming to the skin surface and unstable electrode-skin interface, which may cause an increase in noise and artifact as well as poor signal for brain activity (Chi et al., 2010; Mota et al., 2013; Tautan et al., 2014). Semi-dry electrodes are a combination of both wet and dry electrodes, where electrolyte liquid is slowly released from a reservoir onto the scalp, instead of the use of conductive gels (Li et al., 2017). Compared to wet and dry electrodes, an advantage of the semi-dry electrode is that it does not require manual application of gel and there is still an excellent EEG signal (Li et al., 2016; Wang et al., 2016). Li et al. (2017) evaluated the effects of type of electrode, skin location, skin abrasion, contact area of dry electrodes, and EEG signal in relation to electrode-skin impedances and found that (a) impedances of the dry electrodes were at least 50 times larger than those of wet and semi-dry electrodes, which may be due to the small amount of electrolyte at the dry electrode-scalp interface and inability to maintain good contact with the scalp; (b) impedances decreased in the following order: from forearm, scalp, to forehead, for all types of electrodes; (c) impedances of both semi-dry and dry electrodes considerably decreased after skin abrasion; (d) increasing the contact area of the electrode to the scalp can lower impedances; and (e) high-quality EEG recordings were not obtained from dry electrodes when compared to semi-dry and wet electrodes. However, by adding a thin cotton pad wetted with saline, the quality of EEG recording from dry electrodes could be improved to the level of semi-dry electrodes. Taken together, researchers are mindful of how these factors and electrodes may impact the quality of their data.

Because African hair types are naturally curlier and tightly coiled, it affects the ability of researchers to control skin abrasion and contact area between the electrode and scalp for high-quality EEG recordings. Although use of different types of electrodes may seem like a reasonable alternative for Black American participants who have African hair types, Mathewson et al. (2017) found that the dry electrodes had slightly increased baseline EEG recording and ERP noise levels, which led to poorer statistical power and required around five times the number of trials as the wet electrodes. While it is not ideal to have five times as many trials in one paradigm while using dry electrodes, traditional EEG and ERP measures were still observed and replicated when compared to wet conditions. Mathewson et al. (2017) acknowledge that a major benefit of the dry electrode system is the ease and speed of application; however, the setup was particularly time consuming for individuals with very thick hair and characteristics of the target population with such hair, thickness must be considered when using dry electrode systems. This directly impacts the recruitment of Black American participants who naturally have African hair types, and it raises the issue that in addition to increasing the number of trials to reach a level of statistical significance in EEG recording with a dry electrode system, the same benefits of using a dry electrode system do not apply to individuals with African types of hair. Here, we see that alternative methods regarding different types of electrodes to improve EEG data quality are not inclusive for African hair types.

A second method researchers consider as an alternative to skin abrasion and reducing S/N ratio is using high-density EEG recording systems. High-density (HD) EEG recording systems contain a large number of electrodes (e.g., ranging from 64 to 128 electrodes), which provides better spatial resolution compared to low-density (LD) EEG recording systems. Researchers have studied how the effects of LD and HD EEG recordings may impact data quality. A disadvantage of HD EEG recording is increased setup time and increased possibility for electrolyte bridging (i.e., spreading) due to the shorter distances between electrodes (Greischar et al., 2004). Electrolyte spreading occurs during initial positioning of electrodes on the scalp and creates a nearly continuous path of brain activity between electrodes, which results in diminished voltage differences between affected electrodes and decrease in S/N ratio (Greischar et al., 2004). Additionally, in the Greischar et al. (2004) study, participants included males who had full heads of hair that were generally straight and a length of about 3 to 8 cm compared to females who had hair about 5 cm above shoulder level and hair characteristics included straight, dense, and curlier hair textures. Greischar et al. (2004) found significantly more bridged electrode pairs in female participants compared to male participants, which suggests that hair length may be a factor for increased electrolyte spreading and decreased amplitude voltage when using HD EEG recordings. Also, to note, scalp thickness and skull thickness affect EEG potentials and may cause inaccurate EEG source localization (e.g., attempts to calculate the likely neural source/generator of electrical signals using HD EEG recordings; Cuffin, 1993; Lehtinen et al., 1996). In this case, Black American men who shave their heads resulting in thicker scalps may have lower EEG potentials and provide inaccurate measurement of brain activity in research studies. Because Black American participants’ baldness and voluminous hair characteristics match some of the female participants’ dense and curlier hair textures, this finding adds to the list of ineffective methods of EEG data collection and analysis that prevents inclusivity in the recruitment and retention of Black American participants.

Based on the findings above, EEG researchers struggle to find appropriate and alternative methods to recruit and retain data collected from Black American participants. Within experimental and health research, a disparity exists with the recruitment of minority participants due to barriers such as language, mistrust, and stigma (George et al., 2014). However, after these barriers are addressed within EEG research, factors like impedances and S/N ratio affect the data quality of acceptable EEG recordings. Because hairstyles worn predominantly by Black American participants reduce proper S/N ratio and result in poor conductivity between the electrode and scalp, African hair types may lead to unsatisfactory quality of EEG recordings; and, ultimately, their EEG data recordings often are excluded from the final data analyses. Furthermore, EEG researchers utilize different electrodes and EEG recordings to measure brain activity, such as wet, dry, and semi-dry electrodes and HD/LD EEG recording. These methods currently utilized in EEG research still present challenges regarding inclusivity for African types of hair worn predominantly by Black American participants, further leading to underrepresentation of Black American participants in neuroscience research. Although unintended systemic bias is a rising concern within neuroscience research, there are bioengineering labs that recognize this dilemma and are in the process of creating and investigating new electrodes that may be better suited for participants with African hairstyles and hair types.

## Potential Solutions in EEG Research

One bioengineering lab, in particular, has developed Sevo electrodes, which include an electrode-bearing hair clip that acts as a barrette to separate the hair and hold the electrode firm against the scalp (Etienne et al., 2020). This reduces impedances and improves S/N ratio by more than 10 times compared to clinical standard electrodes (Etienne et al., 2020). To further improve conductivity between the Sevo electrode and the scalp, the research team also prepared their participant’s hair by braiding hair into a simple style of cornrowing referred to as “straight backs,” or braiding hair in different configurations as long as the electrode makes contact with the scalp to allow for the standard positioning of electrodes on a participant’s scalp. Although African hair types negatively impact EEG data quality, Etienne et al. (2020) have turned a traditional African hairstyle into an advantageous, creative solution for improving adherence to the scalp. A third potential solution proposed by Casson (2019) are fingered electrodes, which are electrodes with “fingers,” or prongs, to push apart the hair and make contact with the scalp. Casson (2019) also proposed the use of conformal temporary tattoos, which are non-permanent EEG sensors similar to children’s rub-on tattoos with built-in adhesives to connect directly to the skin, for non-haired regions in EEG for clinic studies. The researchers suggested the use of a hybrid method with both fingered electrodes for haired regions and temporary tattoos for non-haired regions as future *beyond wearable* EEG systems.

Lastly, Sun et al. (2012) tested a new design of the skin screw electrode that can hook painlessly, but mechanically to the strong top layers of the participant’s skin by slightly twisting and pressing and has micro teeth that are angled on the bottom rim of the electrode. This technique makes electrical contact without the need of applying electrolyte (i.e., gel or saline solution) and the hairs of the participant are pressed into the space between the micro teeth, improving contact with the skin. When evaluating the performance of the skin screw electrode, results showed that the skin screw electrode (1) has impedance comparable to standard commercial scalp EEG; (2) provides comparable EEG data quality; and (3) significantly outperforms the commercial scalp EEG in application time and stability. Utilizing these novel, more convenient, and empirically validated non-invasive electrodes, researchers will not only improve quality signal between the scalp and electrode, but it may also increase recruitment and retention for Black American participants who have African hairstyles and hair types.

## Considerations and Potential Pitfalls

As new technologies arise, the feasibility and validity of these electrodes should be considered. First, in regard to the fingered electrodes and conformal temporary tattoos, Casson (2019) recommended performing a range of studies to test the validity of two different types of electrodes by placing them on the head simultaneously, which will allow the two signals to be compared. However, this may be challenging, because EEG varies spatially and brain signals differ by head region. A possible solution may be to place the test electrode closer to the reference electrode in hopes of minimizing the differences between the two electrodes. Second, although Etienne et al. (2020) provided detailed descriptions of how to design and create the Sevo electrode (e.g., hair clip), the cost and feasibility of replicating the engineering of this ingenious solution have yet to be evaluated. Additionally, Sevo electrodes (e.g., an arrangement of 10–20 electrodes) may not be applicable or comparable to high-density EEG (e.g., ranging from 64 to 128 electrodes) preparation and data quality. Etienne et al. (2020) suggested the Sevo electrode is a beneficial technique for clinical patients with seizures; therefore, future research is still needed to test the applicability of the Sevo electrodes with non-clinical populations with similar hairstyles and hair types. It is of note that Sun et al. (2012) have found a simple and cost-effective way to manufacture the electrode teeth using light as a process to create microfabrication, which has been utilized widely in the semiconductor industry. Third, these solutions may not be feasible for all populations. Both studies by Etienne et al. (2020) and Sun et al. (2012) were conducted on healthy adult participants, and neither examined possible issues of preparation for children. Fourth, researchers should take into consideration the preparation of utilizing Sevo electrodes. Not all research staff will know how to cornrow hair into “straight backs,” which effectively reduces impedances and improving S/N ratio, and it may seem intrusive for researchers to braid participants’ hair. Above all, these innovative technologies are predominantly researched and implemented in the realm of bioengineering rather than neuroscience. As a result, there is a gap in the implementation of these inclusive solutions within the field of neuroscience. Because neuroscience researchers do not often have the means to engineer new electrodes, they heavily rely on bioengineers to bridge the gap between innovative techniques and utilization of effective and valid technologies in neuroscience research.

## Conclusions

In summary, the current paper summarizes an unexpected intersectionality between the field of neuroscience and systemic racism. Specifically, Black American participants are often excluded in EEG research due to challenges adapting EEG methodology to account for the variations of African hair types of curly and tightly coiled hair. Because standard quality EEG recording and data analysis require acceptable S/N ratio and proper adherence between the electrode and scalp, African hairstyles and types of hair may prevent good conductance of brain signal and impact the accurate measurement of brain activity. Though this impacts recruitment and retention of Black American participants in EEG research, bioengineering labs have recognized the lack of adaptation to measure and include brain data collected from Black American participants within EEG research. They have proposed solutions, such as engineering innovative electrodes to improve adherence between the electrode and scalp and braiding hair in a certain way to maneuver thick and coarse hair and to expose the scalp in areas where the electrode will be adhered.

To our knowledge, this is the first position paper to recognize this growing issue of diversity and neuroscience, while also summarizing potential solutions to improve the inclusivity of Black American participants in EEG research. Note that the proposed solutions are likely not exhaustive, nor do they reflect comprehensive solutions to the issues of systemic bias/racism in neuroscience research. However, these solutions represent important steps towards making neuroscience research more inclusive. It is also important to note that issues related to recruitment and retention of Black American participants in neuroscience research would likely be less prevalent if researchers themselves were representative of the population. That is, these systemic issues in neuroscience likely persist due, in part, to the relative lack of diversity in these professions. Along with potential solutions mentioned above related to EEG methods, it is also important to consider broader issues of inclusivity in the fields of science, technology, engineering, and math (STEM). Beyond the methodological issues described above, which often prevent the inclusion of Black American participants in research, researchers should also be aware of how the lack of diverse samples in neuroscience research may impact our understanding of neural processes in minority individuals, which reduces the generalizability of our findings. These systemic biases may lead to unintended consequences, including lack of theoretical understanding and knowledge of how brain function and behavior are interconnected in minority samples, or lack of standard resources and/or support services for minority populations. Recently, many researchers have recognized these issues and proposed methods to facilitate and increase participation of minority populations in research.

As academic research continues to advance, it is our job as neuroscience researchers to first acknowledge that the field is perpetuating biases by inadvertently excluding minority groups, predominantly Black American participants with African hair types, from research studies. It is our hope that by acknowledging the current systemic biases and unintended exclusions, we can advance the field towards practicing and utilizing innovative ideas that may be more inclusive in the recruitment and retention of minority populations.
